# The Organization of Somatostatin-Immunoreactive Cells in the Visual Cortex of the Gerbil

**DOI:** 10.3390/biomedicines10010092

**Published:** 2022-01-01

**Authors:** Kyung-Min Kwon, Myung-Jun Lee, Han-Saem Chung, Jae-Hong Pak, Chang-Jin Jeon

**Affiliations:** 1 Department of Biology, School of Life Sciences, BK21 FOUR KNU Creative Bio-Research Group, College of Natural Sciences, Brain Science and Engineering Institute, Kyungpook National University, Daegu 41566, Korea; us3469@knu.ac.kr (K.-M.K.); bridlee900@knu.ac.kr (M.-J.L.); 2 Research Institute for Dok-do and Ulleung-do Island, Department of Biology, School of Life Sciences, Kyungpook National University, 80 Daehak-ro, Buk-gu, Daegu 41566, Korea; jhpak@knu.ac.kr; 3 Department of Biology, School of Life Sciences, College of Natural Sciences, Kyungpook National University, Daegu 41566, Korea; 2018117591@knu.ac.kr

**Keywords:** somatostatin, visual cortex, gamma-aminobutyric acid, calcium-binding proteins, calcium/calmodulin-dependent protein kinase II, dopamine receptors, nicotinic cholinergic receptors, Mongolian gerbil, immunocytochemistry

## Abstract

Somatostatin (SST) is widely expressed in the brain and plays various, vital roles involved in neuromodulation. The purpose of this study is to characterize the organization of SST neurons in the Mongolian gerbil visual cortex (VC) using immunocytochemistry, quantitative analysis, and confocal microscopy. As a diurnal animal, the Mongolian gerbil provides us with a different perspective to other commonly used nocturnal rodent models. In this study, SST neurons were located in all layers of the VC except in layer I; they were most common in layer V. Most SST neurons were multipolar round/oval or stellate cells. No pyramidal neurons were found. Moreover, 2-color immunofluorescence revealed that only 33.50%, 24.05%, 16.73%, 0%, and 64.57% of SST neurons contained gamma-aminobutyric acid, calbindin-D28K, calretinin, parvalbumin, and calcium/calmodulin-dependent protein kinase II, respectively. In contrast, neuropeptide Y and nitric oxide synthase were abundantly expressed, with 80.07% and 75.41% in SST neurons, respectively. Our immunocytochemical analyses of SST with D_1_ and D_2_ dopamine receptors and choline acetyltransferase, α_7_ and β_2_ nicotinic acetylcholine receptors suggest that dopaminergic and cholinergic fibers contact some SST neurons. The results showed some distinguishable features of SST neurons and provided some insight into their afferent circuitry in the gerbil VC. These findings may support future studies investigating the role of SST neurons in visual processing.

## 1. Introduction

Somatostatin (SST), first isolated from the ovine hypothalamus in 1973, acts as a growth hormone inhibitory peptide [1]. SST impacts a wide variety of physiological functions, such as hormonal regulation [1,2], gastrointestinal regulation [3], plasticity [4,5,6], learning [5,7], memory [6], and visual processing [8,9,10]. Pathologically, SST is involved in multiple diseases, such as schizophrenia [11], Alzheimer’s disease [6], depression [12], and obesity [13].

The identification of the transmitter-, neuromodulator-, and peptide-specific heterogenous types of neurons is essential to understanding the brain’s functioning. In the cerebral cortex, SST neurons constitute a large cortical subpopulation of interneurons, comprising approximately 30% of gamma-aminobutyric acid (GABA)ergic interneurons [14,15,16]. Three major EF-hand calcium-binding proteins (CBPs), calbindin-D28K (CB), calretinin (CR), and parvalbumin (PV), have been extensively utilized to localize different types of cortical interneurons and SST neurons [17,18,19,20]. Two additional markers have been used to identify interneuron subtypes and have been known to have overlapping populations with SST neurons: neuropeptide Y (NPY) and nitric oxide synthase (NOS). NPY, one of the most abundantly expressed neuropeptides in the brain, is mostly seen in GABAergic neurons. NOS produces a retrograde neurotransmitter, nitric oxide [21,22,23,24,25].

Similarly to the somatosensory and auditory cortex, the visual cortex (VC) is composed of six distinct cortical layers that are characterized by their synaptic in- and out-puts and by specific cell types present in each layers [26]. The major class of projection neurons in the VC is the heterogenous types of pyramidal cells which are located in any of the cortical layers except layer I. Extremely large number of genetic, functional and/or structural types of cortical interneurons have been identified [26,27,28,29,30]. In this study, we focused on SST neurons that are specifically found in cortical layers II/III and V/VI. SST neurons have been identified in the VC of various animals including mammals and non-mammals, such as monkeys [31,32], cats [33,34,35,36,37], rabbits [38], rats [31,39,40,41,42,43], mice [14], pigeon visual Wulst [44], and the squid optic lobe [45]. The majority of SST neurons in the mammalian VC are non-pyramidal interneurons [14,32,34,35,39,40,41,46,47]. The cortical distribution of SST neurons varies with animal species, but they are generally concentrated in either layer II/III [32,38,40,42,43] or V/VI [32,34,37,41].

Mongolian gerbils, *Meriones unguiculatus*, are rodents belonging to the subfamily Gerbillinae. Gerbils are widely used as research models in areas such as aging [48], metabolism [49,50,51,52,53,54], anatomy [55,56], and parasitic diseases [57,58]. In neuroscience research, gerbils have been widely used to study sensory systems [59,60,61,62,63], neural diseases [61,64,65,66], and brain structure [67,68,69]. Mice and rats are common in biomedical and neuroscience research due to their genetic manipulability and well-published data. However, gerbils are particularly important and have specific advantages for studies on the central visual system. For example, mice and rats are nocturnal animals with rod-dominated retinae. Gerbils, however, are diurnal animals with a higher proportion of retinal cones compared to mice and rats [70,71,72], thus giving them a higher visual acuity [73]. Furthermore, gerbils have a specialized retinal region, which is similar to the human fovea [72,74]. Accordingly, gerbils have been used widely in studies of the central visual system, such as the retina [71,72,75,76] and sensory cortex, including the VC [61,62,63,77].

Although SST neurons have been extensively examined in various animals, the neuronal architecture of SST neurons has not been studied in the Mongolian gerbil VC. Pursuing this knowledge of such an excellent animal model holds important implications for central visual system research. Therefore, we have designed this study to investigate the organization of SST neurons in the gerbil VC.

It is well known that the morphological identification of neurons is a direct reflection of their functional connection and, thus, provides a fundamental understanding of the brain. Therefore, we first examined the distribution and morphology of SST neurons to identify any heterogenous subtypes. Next, we determined the expression patterns of GABA in SST neurons to determine any species differences. Then, as cell types can be distinguished by the expression of specific peptides, we determined different expression patterns of various CBPs, NPY, and NOS in SST neurons. Finally, although some outputs of SST neurons have been studied in detail, little is known about the synaptic inputs into these cells; hence, we decided to analyze dopaminergic and cholinergic receptors in SST neurons. Both dopamine [78,79] and SST [80] are known to play a role in mood regulation. It is also known that cholinergic signaling through neuronal nicotinic acetylcholine receptors (nAChRs) modulates higher cognitive functions [81], including memory [82]. Interestingly, SST neurons also contribute to cognition [8] and memory formation [6]. Our results show diverse heterogenous types of SST neurons based on varying morphologies and expressional patterns of GABA, CBPs, NPY, and NOS. We also provide evidence to suggest possible connections of SST neurons with dopaminergic and cholinergic neurons.

## 2. Materials and Methods

### 2.1. Animal and Tissue Preparation

A total of 15 adult Mongolian gerbils (*Meriones unguiculatus*) (aged 3–4 months, weighing 70–90 g), obtained from a local vendor, were used in this study. Animals were group housed under a 12 h light:12 h dark cycle until used for the study. The temperature and humidity in the facilities ranged from 23 to 26 °C and from 45 to 65%, respectively. All of the animals were deeply anesthetized with isoflurane (5% in O_2_) and perfused intracardially with 4% paraformaldehyde and 0.3% glutaraldehyde in 0.1 M sodium phosphate buffer (pH 7.4) containing 0.002% CaCl_2_. Procedures for perfusion, isolation of the brain and tissue sectioning were based on those previously described [83,84,85]. The Guide for the Care and Use of Laboratory Animals (https://grants.nih.gov/grants/olaw/guide-for-the-care-and-use-of-laboratory-animals.pdf; accessed on 20 October 2021) was followed. All of the animal experiments were approved by the Animal Care and Use Committee of Kyungpook National University (permission NO. 2014-0181).

### 2.2. Horseradish Peroxidase Staining

Monoclonal rat anti-SST was used as the primary antibody and biotinylated anti-rat IgG produced in goats was used as the secondary antibody (Table 1). Both antibodies were diluted at a ratio of 1:200. Standard immunocytochemical techniques and methods were used, as previously described [83,84]. As a negative control, some sections were incubated in the same solution without the addition of the primary antibody. These control tissues showed no SST immunoreactivity. The final sections were examined and photographed on a Zeiss Axioplan microscope (Carl Zeiss Meditec, Inc., Jena, Germany) with conventional or differential interference contrast (DIC) optics.

### 2.3. Fluorescence Immunocytochemistry

Standard immunocytochemical methods were employed as described earlier [83,84,85]. Cortical sections were double-labeled for SST with each of the following: GABA, CBPs (CB, CR, PV), NPY, NOS, CaMKII, and D_1_ or D_2_ dopamine receptors. Triple-labels were conducted on sections for SST, choline acetyltransferase (ChAT), and α_7_ or β_2_ nAChRs. Labeled sections were preserved under coverslips in Vectashield mounting medium (Vector Laboratories, Inc., Burlingame, CA, USA). The complete information of the primary and secondary antibodies used is listed in Table 1.

### 2.4. Quantitative Analysis

All of the methods have been described in detail elsewhere and are only summarized here [85]. For the quantitative analysis of laminar distribution, a total of 9 sections, with a width of 2000 μm each, were sampled from each of the 3 animals (3 tissue sections per animal). The morphological types were analyzed for 558 neurons from 9 sections in 3 gerbils. The average diameter and area of SST neurons were determined for 202 neurons analyzed from 20 sections in 3 gerbils. Double-labeled neurons were counted from a total of 9 different tissue sections from each of the 3 animals, each 2000 μm in width, across all layers.

### 2.5. Synaptic Identification

Triple-labeling of SST neurons with ChAT-immunoreactive (IR) fibers and nAChRs was captured using a Zeiss LSM800 laser scanning confocal microscope (Carl Zeiss Meditec, Inc.) with a 100× objective. At the contact point between the SST neurons, ChAT-IR fibers, and receptors, z-series images of these 3 components were taken at 0.2 μm intervals along the *z*-axis using the same laser scanning confocal microscope. The images were viewed using an EC Plan-Neofluar 10×, C-Apochromat 40×/1.2 W, and/or 100×/1.2 oil, with objectives at 2.5× zoom. We obtained approximately 70–80 confocal images at the presumed synaptic contact. The z-series of the confocal images were reconstructed as three-dimensional (3D) images using the ZEN imaging software (2.3 blue edition service pack 1, Carl Zeiss Microscopy GmbH, Jena, Germany). The 3D images and orthogonal views (xy, xz, and yz planes) were used to identify points where the ChAT-IR fibers and α_7_ or β_2_ nAChRs contacted the SST neurons.

## 3. Results

### 3.1. Laminar Distribution of SST Neurons

**Figure 1:**
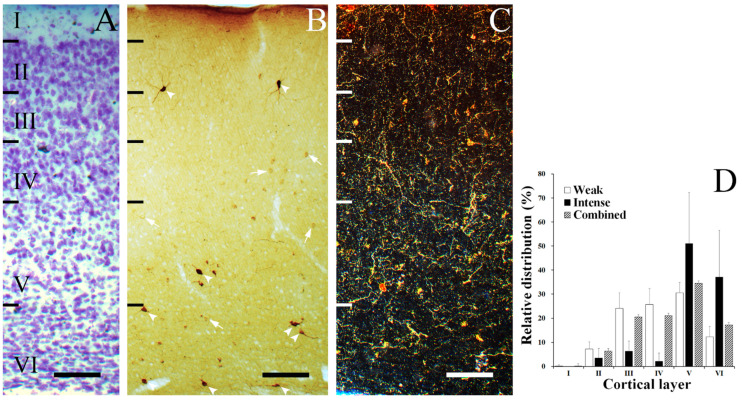
Laminar distribution of SST neurons in the gerbil VC. (**A**) Thionin-stained section illustrating the layer division in gerbil VC. (**B**) Immunostained section showing distribution of SST neurons in gerbil VC. SST neurons in gerbil VC can be distinguished as either intensely (arrowheads) or weakly (arrows) stained, based on their intensity of staining. (**C**) Low-magnification dark field photomicrograph of the gerbil VC with SST fibers dispersed throughout whole layers. (**D**) Histogram of the relative distribution of intensely and weakly stained SST neurons in the gerbil VC. The error bars represent standard deviation (S.D.). Scale bar = 100 μm.

SST neurons were selectively distributed in the gerbil VC (Figure 1). Figure 1A shows a thionin-stained section of a gerbil VC, which revealed division of the cortical layers. Figure 1B shows the laminar distribution of SST neurons with different intensities of staining. The first group of neurons were intensely stained (Figure 1B, arrowheads). These intensely stained neurons were mostly located in layers V–VI and sparsely distributed through II–IV (Figure 1D, black colored bars). The second group of neurons were weakly stained (Figure 1B, arrows), and these types of neurons showed a more even distribution compared to the former group (Figure 1D, white colored bars). In both groups, the distribution peaked at layer V and was absent in layer I, except a single weakly stained SST neuron found in layer I. Figure 1C represents a dark field image of SST fibers forming a plexus throughout multiple layers. Figure 1D shows the relative frequency of the laminar distribution of the two differently stained SST neurons and the sum of both calculated SST neurons, respectively. Quantitatively, 0% ± 0% (mean ± S.D.) of intensely stained SST neurons were found in layer I, 3.50% ± 3.96% were found in layer II, 6.29% ± 4.32% were found in layer III, 2.10% ± 3.47% were found in layer IV, 51.05% ± 21.2% were found in layer V, and 37.06% ± 19.54% were found in layer VI. Furthermore, 0.17% ± 0.54% of weakly stained SST neurons were found in layer I, 7.20% ± 3.03% were found in layer II, 24.01% ± 6.58% were found in layer III, 25.73% ± 6.57% were found in layer IV, 30.53% ± 4.35% were found in layer V, and 12.35% ± 4.34% were found in layer VI. 0.14% ± 0.44% of total SST neurons were found in layer I, 6.47% ± 2.41% were found in layer II, 20.52% ± 8.41% were found in layer III, 21.07% ± 4.71% were found in layer IV, 34.57% ± 4.78% were found in layer V, and 17.22% ± 4.49% were found in layer VI (Figure 1D, striped bars).

### 3.2. Morphology of SST Neurons

**Figure 2:**
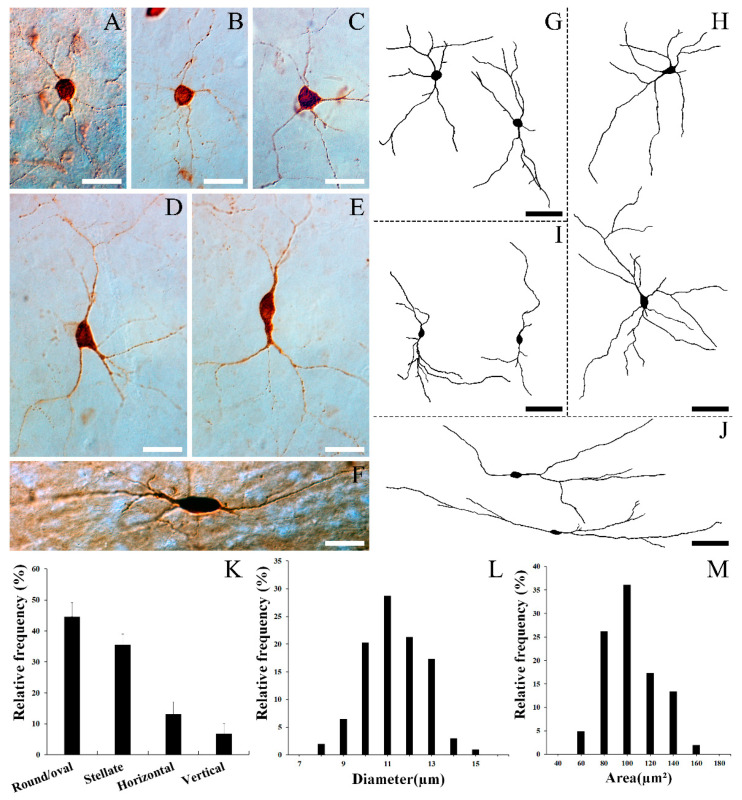
Images of SST neurons’ morphologies and quantitative data in the gerbil VC. DIC photomicrographs (**A**–**F**) and drawings (**G**–**J**). (**A**,**B**,**G**) Multipolar round/oval cell. (**C**,**D**,**H**) Multipolar stellate cell. (**E**,**I**) Vertical fusiform cell. (**F**,**J**) Horizontal cell. Although the drawings are copies based on the best-stained cells, note that cell depictions may not be realistic due to the limitations of immunocytochemistry, such as imperfect filling of the whole cell body and trimming of fibers. (**K**) Histogram of the morphological distribution of SST neurons in the gerbil VC. Multipolar round/oval cells were the most commonly found. The average diameter (**L**) and area (**M**) of SST neurons in the gerbil VC were calculated. The average diameter ranged from 7.50 to 14.48 μm, with a mean of 10.81 μm (S.D. = 1.34). The average area of these cells ranged from 44.20 to 153.12 μm^2^, with a mean of 93 μm^2^ (S.D. = 22.70). The error bars represent standard deviation (S.D.). Scale bar = 20 (**A**–**F**) and 50 μm (**G**–**J**).

Figure 2A–J show the representative form of each cell type. Figure 2A,B,G represent multipolar round/oval cells, the most common type in SST neurons, which have a round/oval-shaped cell body and multiple dendrites extending from the cell body to many directions. The multipolar round/oval cells had medium dendritic fields (200–300 µm in diameter). Figure 2C,D,H show multipolar stellate cells, which are the second most common type among SST neurons. Similarly to round/oval cells, these types of cells also have multiple dendrites proceeding to various directions. However, the polygonal-shaped cell body distinguishes these cells from round/oval cells. In general, the multipolar stellate cells had larger dendritic fields (300–400 µm in diameter) than round/oval cells. Figure 2E,I show typical vertical fusiform cells. These cells have a vertical fusiform cell body with two main processes, each ascending towards the pial surface and descending to lower layers, respectively. These types of cells have medium to large dendritic fields (200–400 µm in diameter). Figure 2F,J are examples of horizontal cells, which have a horizontally oriented fusiform cell body and horizontally oriented long dendrites. In the present study, some fibers in this cell type were found to have more than 500 μm-long fibers (Figure 2J). Although a large amount of the SST-staining in the present study is notable, we still face the high possibility of a lack of complete staining of cells due to the limitations of immunocytochemistry, and truncations of some cell processes due to the sectioning and curving of processes. Thus, the labeling of dendrites was not sufficiently extensive to provide full descriptions of the dendritic size and morphology.

Figure 2K reveals the relative frequency of each cell type in the gerbil VC. Quantitatively, 44.62% ± 3.94% (mean ± S.D.) (249 of 558 cells) of SST neurons were round/oval, 35.48% ± 3.44% (198 of 558 cells) were stellate, 13.08% ± 3.98% (73 of 558 cells) were horizontal, and 6.81% ± 3.09% (38 of 558 cells) were vertical fusiform. Figure 2 shows the average diameter (O) and area (P) of SST neurons, respectively. The average diameter of SST neurons ranged from 7.50 to 14.48 μm, with a mean of 10.81 μm (S.D. = 1.34 μm). The vast majority of SST neurons (87.62%, 177 of 202 cells) had a diameter ranging between 9 to 13 μm, and none of these cells had a diameter > 15 μm. The area of these cells ranged from 44.20 to 153.12 μm^2^, with a mean of 93.00 μm^2^ (S.D. = 22.70 μm^2^).

### 3.3. Colocalization of SST with GABA, CBPs, NOS, NPY, CaMKII, Dopamine Receptors, and ChAT with nAChRs

**Figure 3:**
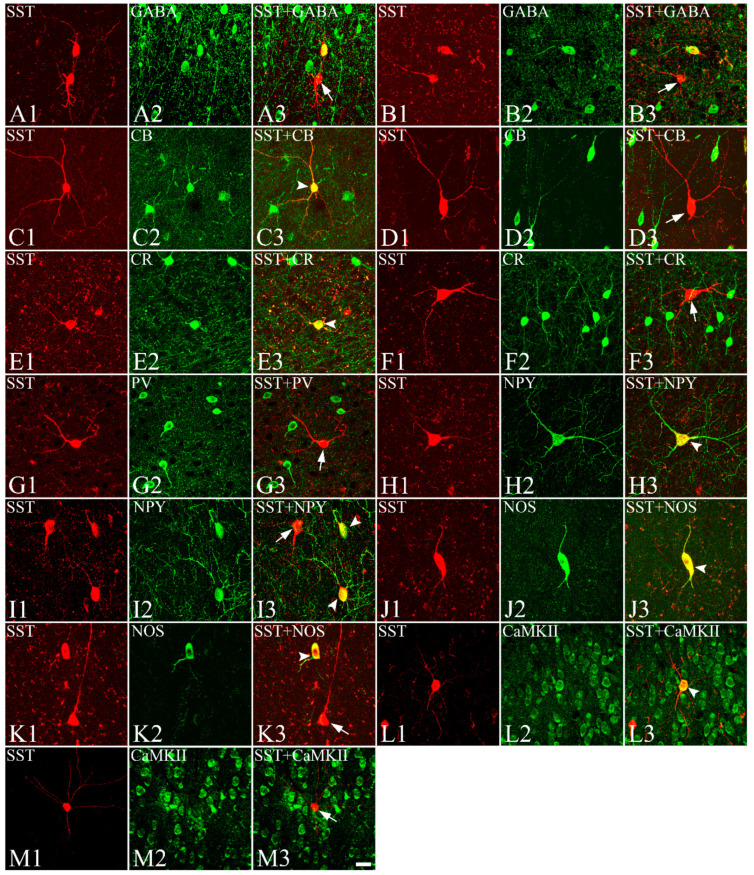
Fluorescence confocal photomicrographs of colocalization of SST neurons (**A1**,**B1**,**C1**,**D1**,**E1**,**F1**,**G1**,**H1**,**I1**,**J1**,**K1**,**L1**,**M1**) labeled with Cy3, shown in red, with GABA- (**A2**,**B2**), CB- (**C2**,**D2**), CR- (**E2**,**F2**), PV- (**G2**), NPY- (**H2**,**I2**), NOS- (**J2**,**K2**), or CaMKII-IR (**L2**,**M2**) neurons labeled with FITC, shown in green, and overlapped images of SST with GABA (**A3**,**B3**), CBPs (**C3**,**D3**,**E3**,**F3**,**G3**), NPY (**H3**,**I3**), NOS (**J3**,**K3**), and CaMKII (**L3**,**M3**) in the gerbil VC. Some of the SST neurons co-expressed GABA (arrowheads in **A3**,**B3**), CB (arrowhead in **C3**), CR (arrowhead in **E3**), NPY (arrowheads in **H3**,**I3**), NOS (arrowheads in **J3**,**K3**), or CaMKII (arrowhead in **L3**). However, none of the SST neurons co-expressed PV (arrow in **G3**). Single-labeled SST neurons were marked with arrows. SST, somatostatin; GABA, gamma-aminobutyric acid; CB, calbindin-D28K; CR, calretinin; PV, parvalbumin; NPY, neuropeptide Y; NOS, nitric oxide synthase; CaMKII, calcium/calmodulin-dependent protein kinase II. Scale bar = 20 μm.

In the present study, we investigated whether the SST neurons in the gerbil VC colocalize with GABA, CBPs, NPY, or NOS. Figure 3 shows neurons stained with SST (Figure 3(A1,B1,C1,D1,E1,F1,G1,H1,I1,J1,K1)), GABA (Figure 3(A2,B2)), CBPs (Figure 3(C2,D2,E2,F2,G2)), NPY (Figure 3(H2,I2)), NOS (Figure 3(J2,K2)), or CaMKII (Figure 3(L2,M2)), and the overlapped images of SST with GABA (Figure 3(A3,B3)), CBPs (Figure 3(C3,D3,E3,F3,G3)), NPY (Figure 3(H3,I3)), NOS (Figure 3(J3,K3)), or CaMKII (Figure 3(L3,M3)). Some cells were clearly labeled with SST and GABA, CB, CR, NPY, NOS, or CaMKII antibodies in the gerbil VC. Other cells were labeled with one of the antibodies, but not both. There was no obvious relationship between cell morphology and whether the cell was single or double-labeled. None of the SST neurons were labeled with PV (Figure 3(G1–G3)). Quantitatively, 33.50% ± 6.04% (66 of 197 cells) of SST neurons were double-labeled with GABA, 24.05% ± 3.73% (51 of 212 cells) with CB, 16.73% ± 2.77% (40 of 239 cells) with CR, 0% (0 of 193 cells) with PV, 75.41% ± 8.64% (200 of 265 cells) with NOS, 80.07% ± 7.80% (217 of 271 cells) with NPY, and 64.57% ± 8.77% (164 of 254 cells) with CaMKII. The percentage of double-labeled cells was relatively consistent across sections and among animals (Table 2).

**Figure 4:**
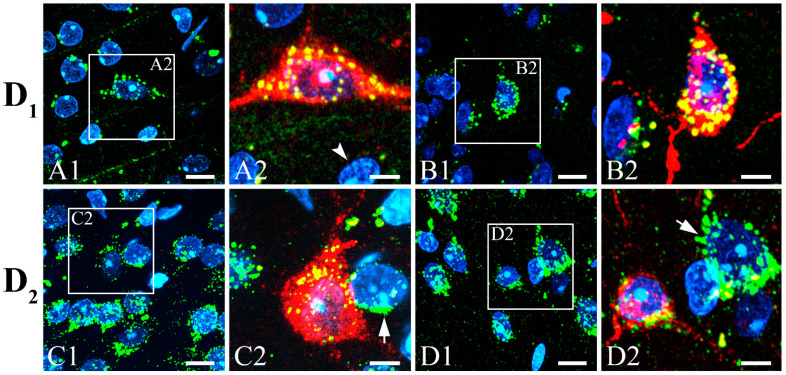
Double-labeling of SST with D_1_ or D_2_ dopamine receptors in gerbil VC. Fluorescence photomicrographs of immunostained D_1_ (**A**,**B**) and D_2_ (**C**,**D**) dopamine receptors (green) surround some cell nuclei stained with 4′,6-diamidino-2-phenylindole (DAPI) (blue). (**A2**,**B2**,**C2**,**D2**) Magnification of white boxes (**A1**,**B1**,**C1**,**D1**) showing merged images of cells co-expressing both SST (red) and dopamine receptors. SST neurons were clearly surrounded by immunopuncta of D_1_ (**A2**,**B2**) and D_2_ (**C2**,**D2**) dopamine receptors. There are cells surrounded by dopamine receptors without SST immunoreactivity (arrows). There are also cells that are immunoreactive to neither dopamine receptors nor SST (arrowhead). Scale bar = 10 (**A1**–**D1**) and 5 μm (**A2**–**D2**). D_1_, D_1_ dopamine receptor; D_2_, D_2_ dopamine receptor.

To determine if SST neurons in the gerbil VC receive synaptic inputs from dopaminergic neurons, we double-labeled them with D_1_ or D_2_ dopamine receptors. Figure 4 shows the double-labeling of SST neurons (red) with well-stained immunopuncta of D_1_ or D_2_ dopamine receptors (green). Some neurons were clearly co-labeled with anti-SST and anti-receptor antibodies. The immunopuncta of D_1_ (Figure 4A,B) or D_2_ (Figure 4C,D) dopamine receptors clearly surrounded some SST neurons (D_1_ in Figure 4(A2,B2) or D_2_ in Figure 4(C2,D2)). There were some cells (arrowhead) that were not surrounded with dopamine receptors or labeled with SST. Conversely, some cells (arrows) were surrounded by dopamine receptors but were not labeled with SST.

**Figure 5:**
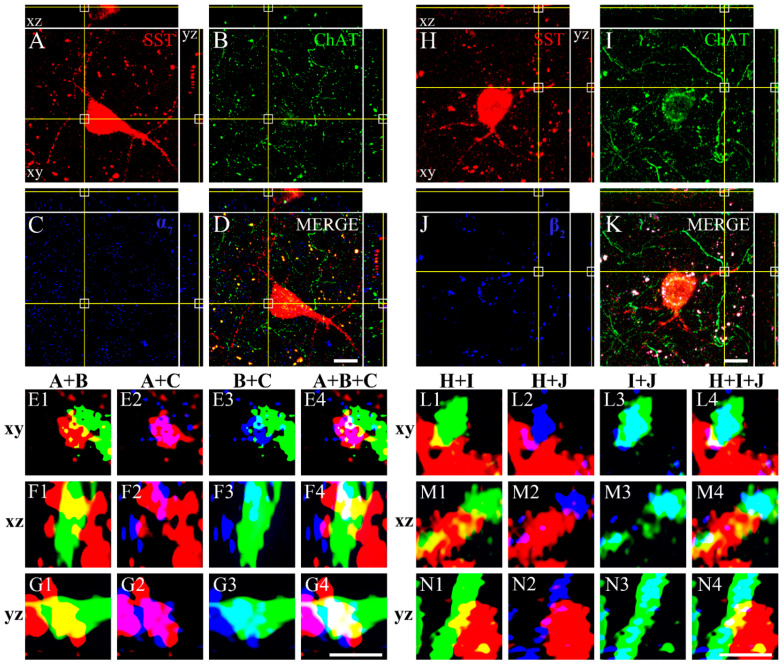
Triple-labeled fluorescence photomicrographs of SST neurons with ChAT-IR fibers and nAChRs. Orthogonal view of a confocal z-stack image of the SST neurons (red, **A**,**H**), ChAT-IR fibers (green, **B**,**I**), α_7_ nAChRs (blue, false color of infrared fluorescence, **C**), merged image of SST neurons with ChAT-IR fibers and α_7_ nAChRs (**D**), β_2_ nAChRs (blue, false color of infrared fluorescence, **J**), and merged image of SST neurons with ChAT-IR fibers and β_2_ nAChRs (**K**). High-power images in crosshair from the xy (**E1**–**E4**), xz (**F1**–**F4**), and yz (**G1**–**G4**) planes from A–D, respectively. High-power images in crosshair from the xy (**L1**–**L4**), xz (**M1**–**M4**), and yz (**N1**–**N4**) planes from H to K, respectively. (**E1**,**F1**,**G1**) Merged images of SST neurons (**A**) and ChAT-IR fibers (**B**) in the xy, xz, and yz planes, respectively. (**E2**,**F2**,**G2**) Merged images of SST neurons and α_7_ nAChRs immunopuncta in the xy, xz, and yz planes, respectively. (**E3**,**F3**,**G3**) Merged images of ChAT-IR fibers and α_7_ nAChRs immunopuncta in the xy, xz, and yz planes, respectively. (**E4**,**F4**,**G4**) Merged images of SST neurons, ChAT-IR fibers, and α_7_ nAChRs immunopuncta in the xy, xz, and yz planes, respectively. (**L1**,**M1**,**N1**) Merged images of SST neurons (**H**) and ChAT-IR fibers (**I**) in the xy, xz, and yz planes, respectively. (**L2**,**M2**,**N2**) Merged images of SST neurons and β_2_ nAChRs immunopuncta in the xy, xz, and yz planes, respectively. (**L3**,**M3**,**N3**) Merged images of ChAT-IR fibers and β_2_ nAChRs immunopuncta in the xy, xz, and yz planes, respectively. (**L4**,**M4**,**N4**) Merged images of SST neurons, ChAT-IR fibers, and β_2_ nAChRs immunopuncta in the xy, xz, and yz planes, respectively. SST, somatostatin; ChAT, choline acetyltransferase; α_7_, α_7_ nicotinic acetylcholine receptor; β_2_, β_2_ nicotinic acetylcholine receptor. Scale bar = 10 (**A**–**D**,**H**–**K**) and 1 μm (**E1**–**G4**,**L1**–**N4**).

In order to identify whether SST neurons in the gerbil VC receive synaptic inputs from cholinergic neurons, we triple-labeled with them ChAT and either α_7_ or β_2_ nAChRs. Figure 5 shows the distribution of SST neurons (Figure 5A,H), ChAT-IR fibers (Figure 5B,I), α_7_ nAChR (Figure 5C), and β_2_ nAChR (Figure 5J). Merged images of the cell with fibers and each receptor are shown in Figure 5D,K. The crosshair reveals the colocalization of SST neurons and ChAT-IR fibers with the nAChRs, in an orthogonal projection. The areas marked with white squares in Figure 5A–D,H–K are displayed at higher magnification in Figure 5(E1–G4,L1–N4). The merged images of SST neurons and ChAT-IR fibers in the xy, xz, and yz planes are shown in Figure 5(E1,F1,G1,L1,M1,N1), respectively. Both SST neurons and ChAT-IR fibers are labeled together in every plane. This indicates the coexistence of the SST neurons and ChAT-IR fibers. Similarly, we identified that SST neurons and α_7_ nAChRs (Figure 5(E2,F2,G2)); ChAT-IR fibers and α_7_ nAChRs (Figure 5(E3,F3,G3)); SST neurons, ChAT-IR fibers, and α_7_ nAChRs (Figure 5(E4,F4,G4)); SST neurons and β_2_ nAChRs (Figure 5(L2,M2,N2)); ChAT-IR fibers and β_2_ nAChRs (Figure 5(L3,M3,N3)); and SST neurons, ChAT-IR fibers, and β_2_ nAChRs (L4,M4,N4) overlap in all 3 planes. These images suggest that cholinergic fibers make synaptic contacts and innervate SST neurons in the gerbil VC.

## 4. Discussion

The present study showed that SST neurons were mainly distributed in layer V of the Mongolian gerbil VC and showed various morphologies. Our results also showed diverse heterogenous types of SST neurons based on the expressional patterns of CBPs, NPY, and NOS. Some SST neurons appeared to be innervated by dopaminergic and cholinergic inputs.

The immunocytochemical labeling of SST neurons showed both intensely and weakly labeled cells. These two labeling patterns represent different distributional characteristics. Intensely labeled SST neurons were highly concentrated in the lower layers, V and VI, of VC. However, weakly labeled neurons were distributed throughout layers III and IV and with a peak at V. Both types of neurons showed almost no labeled cells in layer I. This is highly consistent with other reported animals [40,41,42,43]. Similarly to the present results, previous studies in rat [32,41] and cat [34] VC also showed the prominent distribution of SST neurons on infragranular layers. However, there are differences in distribution patterns among studies with various animals. The majority of SST neurons were in layer II and III in rat [40,42,43], mouse [14], rabbit [38], and monkey [32] VC. In addition, our results showed a relatively large portion of weakly labeled SST neurons in layer IV, which seems to be an unusual pattern compared to other studies, describing the presence of very few cells in layer IV of rat [39,41], cat [34], rabbit [38], and monkey VC [32]. Although there is still no explanation for the differences in distribution between species, these facts might suggest that there are subtle differences in the role of SST neurons in VC between animals.

The cortex contains extremely large numbers of functional and morphological types of neurons [27]. Based on the cortical tiling arguments, hundreds of different cell types have been suggested in the neocortex [28]. Moreover, based on the investigations of functional connectivity revealed by laser scanning photostimulation, at least 156 cell types have been suggested in layer IIIb in the primary VC of macaque monkeys [29]. The morphology of SST neurons in the gerbil VC showed diverse types of non-pyramidal interneurons. The majority of the cells were round/oval cells. The next most common were the stellate cells. Vertical fusiform and horizontal cells were also found in the gerbil VC. As in the present study, SST neurons in rat [39,40,41,46] and monkey VC [32] are non-pyramidal cells with a multipolar, bitufted morphology. The cat VC also contains the same morphological features of SST neurons with multipolar, bitufted, bipolar, and Martinotti cells [34,37]. However, in cats and rabbits, few pyramidal-like cells were observed [35,86]. Taken together, these results show that the morphological shape of SST neurons in VC is in general agreement among animal species. However, the various distinct types of SST neurons reflect the diverse functions and connections with other neurons of SST neurons in VC. It will be very important to elucidate the functions and circuits of each type of SST neurons in the future.

SST neurons in the gerbil VC showed variable sizes but small-to-medium sized cells were most prevalent. No cells bigger than 15 μm were found. Similarly to the present study, the monkey VC also contained small multipolar SST neurons with diameters of 10 to 12 μm. However, contrary to the present study, large multipolar cells of more than 16 μm in diameter and bitufted cells with lengths of 18 to 30 μm have also been described in monkey VC [32]. Small round cell bodies, 8 × 8 μm^2^ in size, and fusiform cell bodies, 20 × 10 μm^2^ in size, have been reported in rat and monkey neocortices, including the VC [31]. Finally, SST neurons in the cat VC were medium-to-large sized (16–30 μm) cells [34,35]. Thus, the sizes of SST neurons between animals are generally inconsistent. The importance of this diversity in size is not yet obvious.

SST neurons are a major group of GABAergic neurons in mammalian cortical areas, including the VC [14]. In the present study, we found that approximately one-third of SST neurons contained GABA. This result is quite contradictory to the fact that SST neurons are thought to be almost GABAergic in VC [34,42]. However, there is a proportion of SST neurons that do not express GABA. Thus, some SST neurons (2–20%) were not labeled with GABA in rat VC [14,39]. In the hippocampus (9%) and entorhinal cortex (18%), various numbers of SST neurons were not labeled with GABA [87,88]. This discrepancy indicates the existence of species diversity and location dependence. Moreover, the presence of GABAergic neurons outside the boundary of interneurons in the mouse neocortex suggests the possibility that unconventional groups of SST neurons could exist [89].

The low rate of GABA expression in SST neurons raises the question of non-GABAergic neuron functions. The excitatory neuronal marker CaMKII was colocalized with many SST neurons in the present study. It is widely agreed that SST mainly acts as an inhibitory neurotransmitter or neuromodulator in the central nervous system. However, there have been reports of the excitatory effects of SST. For example, SST had a potent excitatory effect on the hippocampus [90], and the effects of SST on mammalian cortical neurons in culture were predominantly excitatory [91]. Most (>90%) SST neurons in the Pre-Bötzinger complex of rat medulla contained vesicular glutamate transporter 2 [92]. In the nucleus of the solitary tract of the rats, one-third of SST neurons, which do not express GAD-67, were found to be vesicular glutamate transporter positive [93]. An unpublished article by Cattaneo [94] suggested that striatal SST interneurons expressed mRNAs for both glutamate and GABA vesicular transporter. These results suggest that the actions of SST are both inhibitory and excitatory.

Many cortical interneurons selectively express specific CBPs [20]. In general, CR and PV are known to form non-overlapping populations with SST neurons and have distinct roles [5,95,96,97]. For example, SST neurons did not express CR in the rat VC [41]. However, the mouse VC expressed 34.3% of CR in SST neurons [97]. An overlapping population (30%) of SST neurons with CR-IR neurons across frontal, somatosensory, and VC has also been reported in mice [98]. Similarly, some SST neurons (16.73%) were double-labeled with CR in the gerbil VC. Our study showed distinct populations of SST neurons from PV, which is in agreement with many former studies. For example, none of the SST neurons colocalized with PV in the rat VC [41]. However, a small number (10%) of cells in the somatosensory cortex of rats co-expressed both PV and SST at the mRNA level [99]. CB is known to comprise a large portion of SST neurons in the cortex [41,95,96]. In the rat VC, 86.3% of SST neurons co-expressed CB [41], 85% of SST neurons co-expressed CB in layers II/III, and 92% in layers V and VI [95]. However, only one-fourth of SST neurons colocalized with CB in the gerbil VC. In the mouse cingulate cortex, one-third of SST neurons co-expressed CB in layers II/III [100]. The combined results again indicate the existence of diversity among animal species and subtle differences in roles that SST neuronal subtypes play.

NPY and NOS have been widely used as markers for the classification of neurochemically distinct interneurons in VC [84,85]. The present study shows large numbers of SST neurons co-expressing NPY (80.07%) and NOS (75.41%). Similarly, 97% and 98% of SST neurons expressed NPY and NOS, respectively, in the guinea pig dorsal striatum [101]. In the rat striatum, 78.3% of SST neurons also expressed NPY [102]. However, only 1.7% of SST neurons expressed NOS in the rat VC [41], and 7% of SST neurons express NOS in the mouse hippocampus [103]. Again, the difference in the expression rates of NPY and NOS in SST neurons between animals and regions seems to be clear. However, the present and previous studies suggest that SST neurons can be further subdivided based on their respective abundance and scarcity of various CBPs, NPY, and NOS. These SST neurons subtypes can present diverse physiological relevance.

The dopamine system controls the physiological function of mood. Indeed, depression is characterized by a decreased dopamine level [104]. The level of SST is also closely related to mood symptoms, whereby decreased SST is a pathological feature in depression [105]. Dopamine receptors are distributed throughout the brain [78]. D_1_ [106] and D_2_ [107] dopamine receptors have been found in the VC. Previous studies have shown that a few SST neurons contained detectable D_1_ dopamine receptor mRNA [108] and that dopamine affected the level of SST via D_1_ and D_2_ dopamine receptors in the striatum [109]. D_1_ and D_2_ receptors are present in inhibitory interneurons, including SST neurons in the monkey frontal eye field [110]. However, minimal colabeling between D_1_ dopamine receptor and SST interneurons was shown in the mouse prefrontal cortex [111]. The present study showed the positioning of D_1_ and D_2_ dopamine receptors in SST neurons in the gerbil VC. These results may suggest a neural connection of dopaminergic neurons to the SST neurons in the VC. Previous studies have shown that SST neurons are responsible for the surround suppression of pyramidal cells in VC [112] and can improve the cognitive function of visual perception in the VC via the inhibition of excitatory neurons to PV+ inhibitory neurons [8,113]. Many previous studies have shown that ChAT plays an important modulatory function in cognition and memory through nAChRs. Additionally, various subtypes of nAChRs have been found in the cerebral cortex [114]. SST is also involved in neuroplasticity, such as enhanced ocular dominance plasticity in VC. Furthermore, SST neurons were activated by acetylcholine [115] and nicotinic receptors [4]. The cholinergic facilitation of the lateral inhibition of neighboring pyramidal neurons in the mouse neocortex is mediated by the activation of β_2_ subunit-containing nAChRs that depolarize SST-positive Martinotti cells [116]. The nAChR modulator, the Lynx family, has been found in SST interneurons in the mouse VC [117]. In this study, we found the presence of α_7_ and β_2_ subtypes of nAChRs in the SST neurons. These results suggest an acetylcholine-SST circuit in the VC, and such information will significantly improve our understanding of SST neuron circuits.

There are differences in the organization of the area of VC between diurnal and nocturnal rodents. The percentage of the cortex devoted to area 17 is significantly greater in the diurnal Nile grass rat compared to the nocturnal Norway rat [118]. However, there were no clear cellular composition differences in area 17 between nocturnal and diurnal rats [118]. The squirrel, a highly visual diurnal rodent with a cone-dominated retina, has a larger amount of cortex devoted to VC compared to nocturnal rodents [119]. The connections from the lateral geniculate nucleus to the VC shows that the squirrel has a well-developed retino-geniculo-cortical system compared to that of the laboratory rat [120]. Several response properties of neurons in the squirrel V1 set diurnal squirrels apart from nocturnal rodents. For example, the squirrel has laminar specificity for direction selectivity, which is lacking in nocturnal rats and mice and has fewer orientation-selective cells in V1 than nocturnal mammals [121]. The V1 of the squirrel contains cells that are tuned to high temporal frequencies, reflecting cone-based vision [121]. Thus, the diurnal squirrel V1 has many similar response properties to larger mammals with a well-developed visual system [122]. However, similarly to other less visual rodents, structured functional maps in visual systems, such as columnar organizations or orientation maps, were lacking in the V1, even in more visual diurnal rodents, such as squirrels [123]. Recently, the orientation- and direction-selective neurons and their spatial layout have been characterized in the primary VC of the large diurnal rodent agoutis. Neurons exhibited orientation and direction preferences in agoutis, with a bias for horizontal contours [124]. The aggregate classical receptive field of agoutis was similar to that of cat areas 17 and 18 and was smaller than that of nocturnal rats and mice [124]. However, the response properties, such as orientation and direction selectivity, simple and complex cells, and spatial and temporal tuning, that have been fairly well-documented in diurnal squirrels, have not yet been well-studied in gerbils. Future studies should elucidate these response properties in gerbils to better understand the diurnal gerbil visual system and the diverse functional organization of the rodent VC as cortical functional architecture can vary greatly from species to species [118,123,124].

Studies on the localization of SST in the human brain have been somewhat limited. One study, using immunocytochemistry, found widespread SST neurons with varying morphologies, sizes, and fibers in whole brain regions and forebrain areas [125]. Distinct topographical localizations of two SST receptor (SSTR) subpopulations in the human cortex have been described using radioligands [126]. The SSTR_1_ was preferentially localized in layers V and VI, while SSTR_2_ was concentrated in the superficial cortical layers (I–IV) and particularly enriched in parts of the lamina IV. The SSTR_4_ localization in the human brain has also been found to include the VC [127]. Both cell bodies and fibers existed at layers III–VI—abundant in layer III—and cell bodies were lacking in I–II. SST mRNA-containing neurons were widely distributed in several areas of the human brain, including the VC [128]. Neurons containing SSTR mRNA also localized in the human cortex, which identified the noticeable distribution of SSTR_3_ mRNA in layers IVc and V of the VC [129]. In the human neocortex, it has been found that the depolarization of SST-positive Martinotti cells by acetylcholine mediates the cholinergic facilitation of lateral inhibition [116]. These results strongly support the evidence that SST neurons exist in the human VC. However, there have been no detailed studies on cell morphology, laminar distribution, or neural networks of SST neurons in the human VC comparable with the present result. Studies in animals are often closely related to humans, but in-depth research on the human VC will be essential for a clear understanding of the organization and function of SST neurons in humans.

The dysfunction of SST in the brain is closely linked to various human neuropsychiatric and neurodegenerative disorders. Patients with major depressive disorder show decreased SST levels [12]. Other neuropsychiatric disorders, such as schizophrenia and bipolar disorder, showed a reduction in SST gene expression and fewer SST-expressing neurons in various brain areas [11,130]. Neurodegenerative disorders, such as Alzheimer’s and Parkinson’s disease, are also closely linked to reduced levels of SST in cortical and subcortical regions [6,80]. Many aspects of the anatomical, physiological, pharmacological, and genetic characteristics of SST neurons remain unknown. However, the attribution of SST in the control of various human brain diseases demands in-depth information, such as specific laminar distribution, distinct cell types, and specific cortical wiring patterns of SST neurons.

## 5. Conclusions

SST neurons included a highly diverse group of interneurons both morphologically and with respect to the presence and absence of GABA, CB, CR, PV, NPY, NOS, and CaMKII implying diverse functional roles. The present study suggests that SST neurons in the VC receive inputs from dopamine and acetylcholine at least via D_1_ and D_2_ dopamine receptors and α_7_ and β_2_ nAChRs for wiring of VC. It will be essential to compare the present study with molecular, physiological, pharmacological, and genetic levels to enhance the understanding of SST function in the VC. With this knowledge, links can be made between SST function and SST-related neurological disorders in humans.

## Figures and Tables

**Table 1:** List of primary and secondary antibodies and their dilution used in present study.

Primary	Type	Dilution		Manufacturer
SST	RtM	1:200		Millipore, Burlington, MA, USA
GABA	MM	1:500		Millipore
CB	MM	1:500		Sigma-Aldrich, Saint Louis, MO, USA
CR	MM	1:500		Sigma-Aldrich
CR	RbP	1:100		Sigma-Aldrich
PV	MM	1:500–1000		Millipore
NPY	RbP	1:500		Immunostar, Hudson, WI, USA
NOS	MM	1:200		BD Biosciences, San Jose, CA, USA
CaMKII	RbP	1:500		Proteintech, Rosemont, IL, USA
D_1_	MM	1:200		Santa Cruz Biotechnology, Inc., Dallas, TX, USA
D_2_	MM	1:200		Santa Cruz Biotechnology, Inc.
ChAT	MM	1:250		Millipore
α_7_	RbP	1:200		Santa Cruz Biotechnology, Inc.
β_2_	RbP	1:200		Santa Cruz Biotechnology, Inc.
**Secondary**	**Conjugation**	**Dilution**	**Target**	**Manufacturer**
HRP				
Goat anti-rat IgG	Biotinylated	1:200	SST	Vector laboratories, Inc., Burlingame, CA, USA
Fluorescence				
Goat anti-rat IgG	Cy3	1:200	SST	Jackson ImmunoResearch Laboratories, Inc., West Grove, PA, USA
Horse anti-mouse IgG	FITC	1:200	CB, CR(MM), PV, GABA, ChAT, D_1_, D_2_, NOS	Vector laboratories, Inc.
Goat anti-rabbit IgG	FITC	1:200	CR(RbP), NPY, CaMKII	Jackson ImmunoResearch Laboratories, Inc.
Goat anti-rabbit IgG	Cy5	1:200	α_7_, β_2_	Jackson ImmunoResearch Laboratories, Inc.

SST, somatostatin; GABA, gamma-aminobutyric acid; CB, calbindin-D28K; CR, calretinin; PV, parvalbumin; NPY, neuropeptide Y; NOS, nitric oxide synthase; CaMKII, calcium/calmodulin-dependent protein kinase II; D_1_, D_1_ dopamine receptor; D_2_, D_2_ dopamine receptor; ChAT, choline acetyltransferase; α_7_, α_7_ nicotinic acetylcholine receptor; β_2_, β_2_ nicotinic acetylcholine receptor; RtM, rat monoclonal; MM, mouse monoclonal; RbP, rabbit polyclonal.

**Table 2:** Percentage of SST neurons, and neurons double-labeled with GABA, CBPs, NPY, NOS, or CaMKII in the gerbil VC.

Antibodies	Animal	No. Sections	No. SST Cells	No. Double	% Double (Mean ± S.D.)
GABA	#1	3	70	23	32.85 ± 5.59
	#2	3	73	23	31.50 ± 3.38
	#3	3	54	20	37.03 ± 6.75
GABA total		9	197	66	33.50 ± 6.04
CB	#1	3	78	19	24.35 ± 0.56
	#2	3	63	16	25.39 ± 1.55
	#3	3	72	16	22.22 ± 5.69
CB total		9	212	51	24.05 ± 3.73
CR	#1	3	58	9	15.51 ± 4.29
	#2	3	84	14	16.66 ± 0.56
	#3	3	97	17	17.52 ± 0.65
CR total		9	239	40	16.73 ± 2.77
PV	#1	3	67	0	0
	#2	3	65	0	0
	#3	3	61	0	0
PV total		9	193	0	0
NPY	#1	3	84	71	84.52 ± 3.73
	#2	3	103	73	70.87 ± 2.49
	#3	3	84	73	86.90 ± 3.47
NPY total		9	271	217	80.07 ± 7.80
NOS	#1	3	68	55	80.88 ± 2.40
	#2	3	103	73	67.20 ± 5.71
	#3	3	84	73	84.72 ± 5.42
NOS total		9	265	200	75.41 ± 8.64
CaMKII	#1	3	86	54	62.79 ± 8.53
	#2	3	85	50	58.82 ± 5.11
	#3	3	83	60	72.29 ± 6.03
CaMKII total		9	254	164	64.57 ± 8.77

GABA, gamma-aminobutyric acid; CB, calbindin-D28K; CR, calretinin; PV, parvalbumin; NPY, neuropeptide Y; NOS, nitric oxide synthase; CaMKII, calcium/calmodulin-dependent protein kinase II.

## Data Availability

Any additional data will be available upon request to the corresponding author.
